# Inhibition of Receptor-Interacting Protein Kinase 1 with Necrostatin–1s ameliorates disease progression in elastase-induced mouse abdominal aortic aneurysm model

**DOI:** 10.1038/srep42159

**Published:** 2017-02-10

**Authors:** Qiwei Wang, Ting Zhou, Zhenjie Liu, Jun Ren, Noel Phan, Kartik Gupta, Danielle M. Stewart, Stephanie Morgan, Carmel Assa, K. Craig Kent, Bo Liu

**Affiliations:** 1 Department of Surgery, School of Medicine and Public Health, University of Wisconsin, Madison, Wisconsin, USA; 2 Department of Vascular Surgery, 2nd Affiliated Hospital School of Medicine, Zhejiang University, Zhejiang, China

## Abstract

Abdominal aortic aneurysm (AAA) is a common aortic disease with a progressive nature. There is no approved pharmacological treatment to effectively slow aneurysm growth or prevent rupture. Necroptosis is a form of programmed necrosis that is regulated by receptor-interacting protein kinases (RIPs). We have recently demonstrated that the lack of RIP3 in mice prevented aneurysm formation. The goal of the current study is to test whether perturbing necroptosis affects progression of existing aneurysm using the RIP1 inhibitors Necrostatin-1 (Nec-1) and an optimized form of Nec-1, 7-Cl-O-Nec-1 (Nec-1s). Seven days after aneurysm induction by elastase perfusion, mice were randomly administered DMSO, Nec-1 (3.2 mg/kg/day) and Nec-1s (1.6 mg/kg/day) via intraperitoneal injection. Upon sacrifice on day 14 postaneurysm induction, the aortic expansion in the Nec-1s group (64.12 ± 4.80%) was significantly smaller than that of the DMSO group (172.80 ± 13.68%) (*P* < 0.05). The mean aortic diameter of Nec-1 treated mice appeared to be smaller (121.60 ± 10.40%) than the DMSO group, though the difference was not statistically significant (*P* = 0.1). Histologically, the aortic structure of Nec-1s-treated mice appeared normal, with continuous and organized elastin laminae and abundant αActin-expressing SMCs. Moreover, Nect-1s treatment diminished macrophage infiltration and MMP9 accumulation and increased aortic levels of tropoelastin and lysyl oxidase. Together, our data suggest that pharmacological inhibition of necroptosis with Nec-1s stabilizes pre-existing aneurysms by diminishing inflammation and promoting connective tissue repair.

Abdominal aortic aneurysm (AAA), which refers to the progressive weakening and dilatation of the infrarenal segment of the aorta, is a common aortic disease associated with high lethality^1^,^2^. The mortality rate of ruptured AAAs is 85 to 90%^3^,^4^. Although small aneurysms do occasionally rupture, the risk of rupture is found to be associated with larger aneurysm size^4^. Currently, surgical interventions, including open surgical and endovascular aneurysm repairs, are the only effective treatments to prevent rupture in patients with AAAs larger than 5.5 cm (male) or 5.0 cm (female)^4^. Small aneurysms (<3.5 cm in diameter)^5^, however, represent the majority (90%) of AAA cases identified by diagnostic screening. Since median aneurysm growth rate is about 0.26 cm/year^6^, the time between diagnosis and surgical intervention often exceeds 7 years. Therefore, establishing pharmacological treatments for small AAAs is both meaningful and urgent.

MMP inhibition is the most extensively studied anti-aneurysm strategy thus far and has advanced to human clinical trials for its potential to treat small aneurysms^7^,^8^. However, a clinical trial conducted by Meijer and colleagues found that the MMP inhibitor doxycycline failed to stabilize small AAAs and did not alter the eventual need for surgical AAA repair^8^. Other experimental strategies, mostly targeting inflammation such as deletion of mast cells, TNFα, CCR2 or IL-1β, also render aneurysm resistance in pre-clinical studies^9^,^10^,^11^,^12^,^13^,^14^. Several anti-inflammatory therapies are currently being evaluated in clinical trails, including, but not limited to, Canakinumab (ACZ885, a human monoclonal antibody targeting IL-1β, ClinicalTrials.gov numbers: NCT02007252) and Ticagrelor (a platelet aggregation inhibitor, NCT02070653).

We searched for additional therapeutic targets by focusing on the depletion of smooth muscle cells (SMCs), a major pathological characteristic of AAA, along with matrix degradation and vascular inflammation^15^,^16^,^17^. Evidence of SMC apoptosis has long been documented in both human and experimental aneurysm tissues^18^,^19^. Using a pan caspase inhibitor Q-VD-Oph in Angiotensin II (Ang II)-induced AAAs in apolipoprotein E (apoE) knockout mice, we showed that SMC apoptosis is essential for aneurysm formation but not required for aneurysm growth^17^. More recently, we demonstrated that necroptosis, a programmed form of primary necrosis, also contributes to aortic SMC depletion in aneurysm^20^. Necroptosis differs from apoptosis morphologically and mechanistically. While apoptosis is regulated by caspases, necroptosis is modulated by receptor-interacting serine/threonine-protein kinase 3 (RIP3) and the closely related kinase RIP1^21^,^22^. Levels of RIP3 protein and mRNA are elevated in aortic SMCs of human and mouse aneurysmal tissues. Mice lacking one or both copies of the *Rip3* gene are resistant to aneurysm induction, at least in the elastase model^20^.

To our knowledge, necroptosis triggered by most stimuli requires the kinase activity of RIP1 and RIP3. Phosphorylation-dependent formation and stabilization of the necrosome which contains RIP1 and RIP3 is an essential signaling step leading to necroptosis^23^. RIP1 is not necessary for all forms of necorptosis^24^,^25^, under certain conditions, this kinase has been shown to be essential for apoptosis^26^. In contrast to the viable and fertile RIP3 deficient mice^27^, *Rip1* knockout mice die shortly after birth^28^. Through targeted disruption of the rip1 gene, Kelliher *et al*. demonstrated an anti-apoptosis function of RIP1 in neonatal lymphoid and adipose tissues presumably due to a failure to activate the transcription factor NF-κB^28^. The divergent developmental functions of RIP1 and RIP3 suggest that these two closely related kinases may not necessarily work in parallel in disease processes.

We have previsouly reported that the protein level of RIP1, along with RIP3, is elevated in human aneurysmal aortic tissues^20^. In the same study, we also analyzed the mouse aortic tissues transiently perfused with elastase (aneurysm group) or heat-inactivated elastase (control group). Compared to the control aortic tissues, aneurysmal aortas contained higher levels of *Rip1 mRNA* than the control aortic tissues^20^. Since *Rip1*^−/−^ mice die perinatally, to study RIP1 in AAA, we turned to a group of anti-necroptosis small molecules represented by necrostatin-1 (Nec-1) that were discovered from a cell-based screen for chemical inhibitors of necroptosis in monocytic U932 cells^29^. Follow-up studies demonstrated that Nec-1 blocks necroptosis by inhibiting RIP1 kinase activity^21^. Although *Rip1* gene deletion is lethal^28^, inhibition of RIP1 kinase by administration of Nec-1 to adult mice is safe and has been employed in many animal models of human diseases including ischemic brain injury^29^, photoreceptor loss^30^, and myocardial infarction^31^. In this study, we tested the hypothesis that inhibition of RIP1 attenuates AAA development and progression using Nec-1 and its improved form called Nec-1s in a murine elastase model. Our data demonstrate that RIP1 kinase plays an essential role in aneurysm pathogenesis. Administration of Nec-1s stabilizes pre-existing small aneurysms in mice. Inhibiting RIP1 after aneurysm induction not only diminishes SMC death and local vascular inflammation, but also promotes tissue repair mechanisms.

## Results

### Necrostatin-1 inhibits smooth muscle cell necroptosis *in vitro* and in elastase-induced murine AAAs

**Figure 1:**
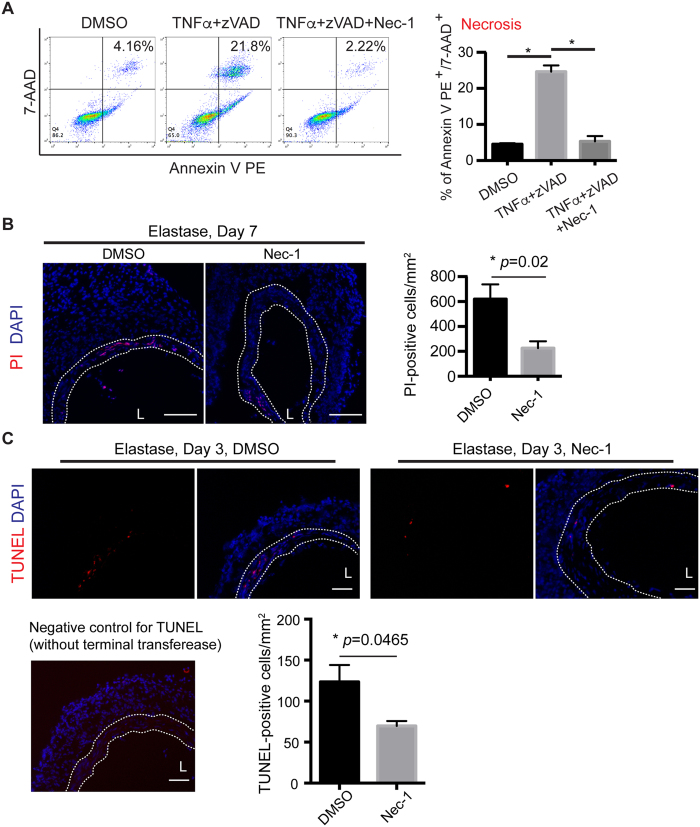
Necrostatin-1 (Nec-1) inhibits SMC necroptosis *in vitro* and attenuates cell death in the elastase-injured aortae. (**A**) MOVAS cells (a mouse aortic smooth muscle cell line) were treated with TNF-α (50 ng/ml), zVAD (40 μΜ), Nec-1 (40 μΜ) or DMSO (vehicle control) as indicated for 6 hours. Cells were then stained with PE Annexin V and 7-AAD and analyzed by flow cytometry. Necrotic cells were identified as PE Annexin V+/7-AAD+. Data represent mean ± SEM. n = 6. *P < 0.05. (**B**,**C**) Representative photographs of aortic sections from DMSO or Nec-1 (3.2 mg/kg/day) treated mice. (**B**) Mice were injected with propidium iodide (PI) 2 hours before euthanization. DAPI was used to stain nuclei. Quantification of PI-positive cells in the media layer (indicated by white dashed line) is shown on the right. (**C**) Apoptotic cells were stained by terminal deoxynucleotidyl transferase dUTP nick end labeling (TUNEL). Bottom left: a representative image of TUNEL staining negative control in which the aortic section was incubated with label solution only without terminal transferase. Bottom right: a bar graph showing quantification of apoptosis (TUNEL-positive) in the media layer (indicated by white dashed line). At least five non-serial cross-sections per aorta were analyzed (n = 3 aortae per treatment). Data represent mean ± SEM. L indicates lumen. Scale bars = 100 μm (**B**), 50 μm (**C**).

We first tested whether RIP1 is required for necroptosis of vascular SMCs. Aortic SMCs were subjected to TNFα (50 ng/ml) and a pan-caspase inhibitor carbobenzoxy-valyl-alanyl-aspartyl-[O-methyl]- fluoromethylketone (zVAD; 40 μM), a commonly used protocol to elicit necroptosis^20^,^23^. As shown in Fig. 1A, TNFα plus zVAD significantly increased the population of cells positive for both Annexin V and 7-AAD (TNFα + zVAD = 24.63 ± 1.73% vs. DMSO control = 4.54 ± 0.27%). Nec-1 (40 μM) completely eliminated the necroptosis response (5.31 ± 1.48%). We defined those TNFα + zVAD induced Annexin V and 7AAD positive cells as necroptotic cells because the cell death is dependent on RIP3^20^.

We next sought to test the anti-necroptosis effects of Nec-1 in a mouse aneurysm model. AAA was induced in C57BL/6J mice by transient perfusion of infrarenal aortae with porcine pancreatic elastase^19^,^32^. Nec-1 (3.2 mg/kg/day) was administered via intraperitoneal (IP) injection 30 minutes before aneurysm induction and injected every day thereafter until sacrifice. Due to the lack of specific biochemical markers for *in situ* identification of necroptosis^20^,^30^,^33^, we and other investigators assess necrotic cells using propidium iodide. Consistent with what we have previously shown^20^, abundant PI-positive necrotic cells were detected primarily in the SMC-rich medial layer (highlighted in white dashed line) of the elastase-injured aortae. Nec-1 markedly reduced the number of necrotic cells compared to the DMSO control (PI-positive cells/mm^2^, Nec-1 = 226 ± 55 *vs.* DMSO = 620 ± 118, *P* = 0.0215, Fig. 1B). We also compared apoptosis between Nec-1 treated aortae and control arteries since we have recently found *in vitro* that RIP1 is involved in macrophage-induced SMC apoptosis^34^. As shown in Fig. 1C, Nec-1 significantly reduced the number of apoptotic cell in the medial layer of elastase-treated arteries (TUNEL-positive cells/mm^2^, Nec-1 = 70 ± 6 *vs.* DMSO = 123 ± 21, *P* = 0.0465).

### Nec-1 prevents elastase and angiotensin II-induced AAA formation in mice

**Figure 2:**
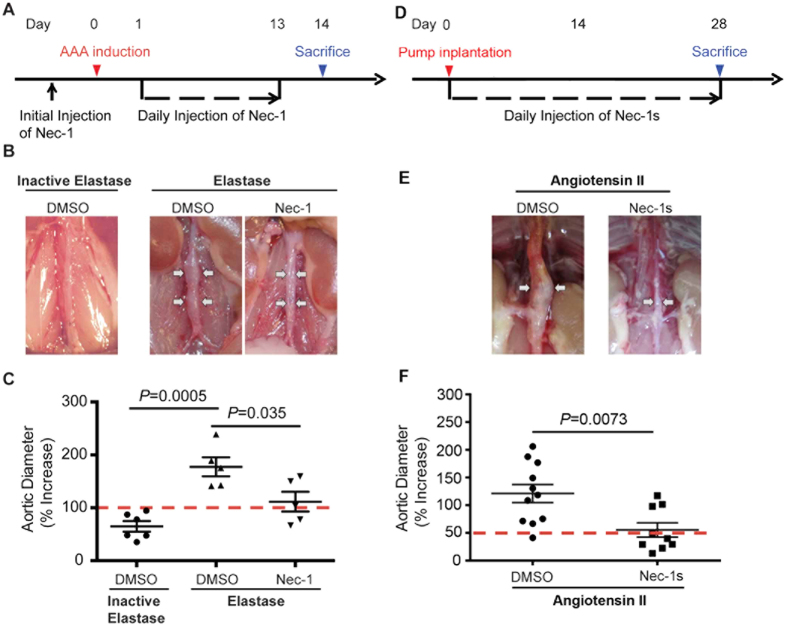
Necrostatin-1 (Nec-1) prevents aneurysm formation in mouse models of aneurysms. **(A**) Experimental design for the elastase model. Mice were treated with vehicle (DMSO) or Nec-1 at 3.2 mg/kg/day starting 30 minutes before elastase perfusion. Mice were euthanized 14 days after. (**B**) Representative photos of perfused abdominal aortae with indicated treatments. Arrows indicate aneurysm formation. (**C**) Percentage increase of maximal external aortic diameter between pre-perfusion and on day 14 after perfusion. In the elastase model, an AAA is defined as a percentage increase in aortic diameter ≥100% (red dashed line). (**D**) Experimental design for the Angiotensin II (AngII) model. *ApoE*^−/−^ mice received osmotic pumps that disbursed Ang II at 1000 ng/kg/min. Daily injection of DMSO or Nec-1s (1.6 mg/kg/day) started immediately following the pump implantation. Mice were euthanized 28 days after. (**E**) Representative photos of abdominal aortae from Ang II-treated mice on day 28. Arrows indicate aneurysm formation. (**F**) Aortic dilatation was expressed as the percentage increase of suprarenal diameter over infrarenal diameter. An AAA is defined in the Ang II model as a ≥50% increase in aortic diameter (red dashed line). Data represent mean ± SEM. One-Way ANOVA and unpaired student’s *t* test were used for C and F, respectively.

Having confirmed the protective property of Nec-1 against both necrosis and apoptosis *in vivo*, we next evaluated the effects of Nec-1 on aneurysm formation in mice by daily IP injections of Nec-1 (3.2 mg/kg/day), beginning 30 min before AAA induction (Fig. 2A). Fourteen days after elastase perfusion, all 5 mice in the DMSO-treated group formed aneurysm (Fig. 2B,C), defined as a 100% or more increase in the maximal external aortic diameter compared to that measured before perfusion^19^. In contrast, in the Nec-1-treated group only 3 of the 5 mice formed aneurysm in response to elastase perfusion. Moreover, Nec-1 significantly reduced the extent of aortic expansion (Nec-1: 111.5 ± 18.67% or 1.142 ± 0.101mm *vs*. DMSO: 177.4 ± 17.96% or 1.502 ± 0.093mm; *P* < 0.05; Fig. 2C and Supplementary Fig. S1A). Heat-inactivated elastase was used as the negative control as it did not produce aneurysmal dilation (Fig. 2B,C). We next investigated the role of RIP1 in aneurysm using another model in which aortic aneurysm formation in hypercholesterolemic *Apoe*^−/−^ mice is induced by angiotensin II (Ang II) infusion for 28 days. Nec-1s (1.6 mg/kg/day) or DMSO (equal volume) were administrated via daily intraperitoneal injection to *Apoe*^−/−^ mice immediately following pump implantation (Fig. 2D). Compared to the DMSO group, Nec-1s-treated mice showed significantly alleviated aneurysm formation (Fig. 2D–F and Supplementary Fig. S1B), reflected by a much smaller aortic dilatation (Nec-1s: 55.40 ± 13.08% *vs*. DMSO: 121.1 ± 16.44%, *P* < 0.05) as well as a reduced AAA incidence (from 90.5% to 33.3%) (Supplementary Table S1).

**Figure 3:**
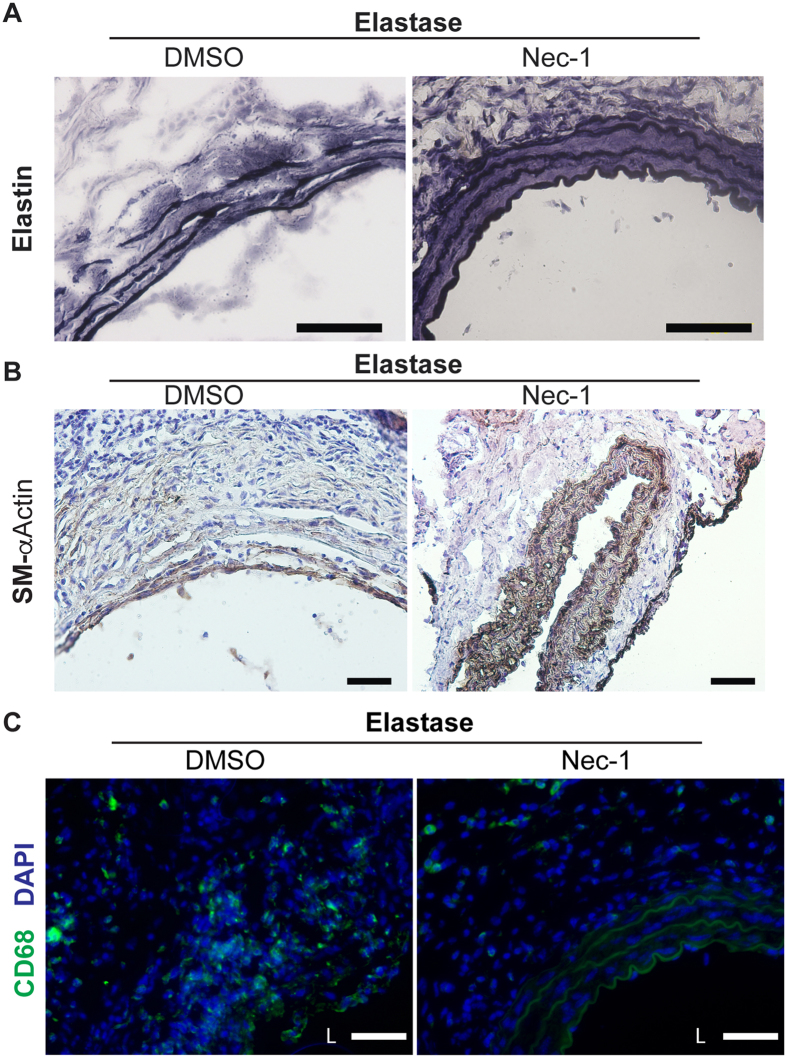
Necrostatin-1 (Nec-1) prevents aortic degradation and inflammation associated with the elastase model. Elastase-perfused aortae were harvested from mice treated with DMSO or 3.2 mg/kg/day Nec-1 on day 14 after surgery. (**A**) Aortic sections were stained with Verhoeff’s working elastic stain solution for elastic fibers which appear black. (**B,C**) Representative photographs of immunostaining for a smooth muscle cell marker SM-αActin (**B**) or for a macrophage marker CD68 (**C**) in the elastase-perfused aortae. DAPI was used to stain the nuclei (**C**). L indicates lumen. Scale bars = 50 μm.

**Figure 4:**
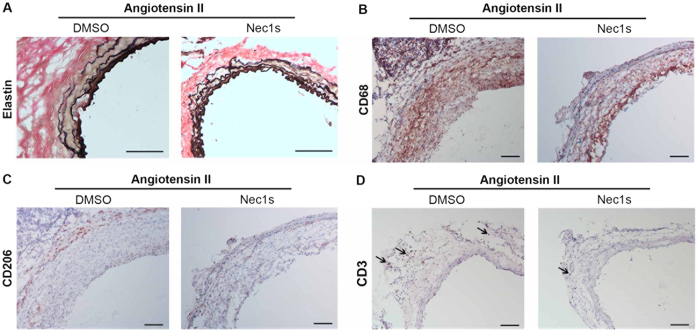
Necrostatin-1s (Nec-1s) prevents angiotensin II induced elastin degradation and aortic inflammation. Aneurysm-prone aortae were harvested from mice treated with DMSO or 1.6 mg/kg/day Nec-1s 28 days after pump implantation. (**A**) Aortic sections were stained with Verhoeff’s working elastic stain solution for elastic fibers (black). (**B–D**) Representative photographs of immunostaining with antibodies specific to CD68 (**B**), CD206 (**C**) or CD3 (**D**). Arrows indicate representative staining of CD3. Scale bars = 100 μm.

We next investigated whether Nec-1 protects mice from developing key pathological features of aneurysms, including disruption of elastin fibers, the loss of aortic SMCs and infiltration of imflammatory cells. Histological analyses showed that elastin fibers in Nec-1-treated aortae appeared continuous and organized, whereas elastin fibers in aortae from DMSO-treated mice were fragmented and disoriented (Figs 3A and 4A). Expression of smooth muscle–specific α-actin (SM-αActin) decreased in AAA tissues (Fig. 3B), reflecting smooth muscle depetion^18^,^20^ as well as smooth muscle phenotypic modulation^35^. In contrast, Nec-1 treatment preserved expression of the SMC marker gene in elastase-perfused aortae (Fig. 3B). Macrophage infiltration into the aorta is another prominent pathological feature of aneurysms^19^,^36^. Compared to the DMSO control, Nec-1 profoundly reduced the number of macrophages accumulated in aneurysm-prone aortae (Figs 3C and 4B). We have previously shown that accumulation of macrophages in the medial layer coincides with SMC apoptosis in elastase-induced mouse AAAs^34^. This association is validated here as Nec-1, which reduced apoptosis in elastase-induced AAAs (Fig. 1C), also diminished the presence of CD68+ positive macrophages in the SMC-rich medial layer (Figs 3C and 4B). We also detected abundant of CD206+ (a marker for M2 or anti-inflmmatory macrophages^37^) cells in the aneurysm-prone aorta. Interestingly, Nec-1s treatment increased the abundance of CD206+ relative to CD68+ cells (Fig. 4C). Moreover, as shown in the new Fig. 4D, we also observed a number of CD3 positive T cells in aneurysms from DMSO group and Nec-1s treatment decreased the infiltration of T cells in the aneurysmal tissues.

### Inhibition of RIP1 blocks progression of existing aneurysms

Most AAA cases are below the threshold for surgical interventions at the time of detection^1^. Therefore, we sought to investigate the possibility that RIP1 inhibition is efficacious for stabilizing existing AAAs. 7 days post-elastase perfusion was selected as the time point to start the RIP1 inhibitor treatment. On day 7, the mean aortic expansion was 100.70 ± 4.37%, around the threshold for aneurysm, defined in the elastase-perfusion model as a ≥ 100% increase in aortic diameter^19^.

**Figure 5:**
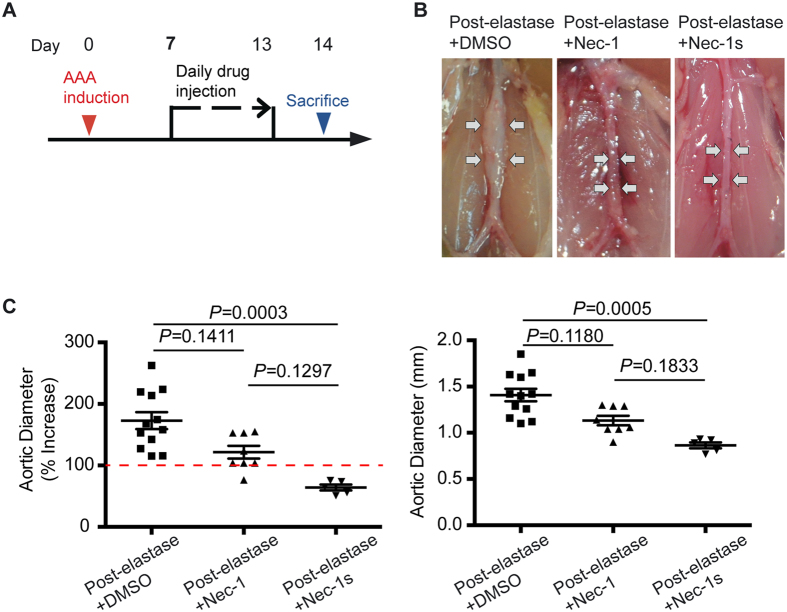
Inhibition of RIP1 kinase with Necrostatin-1s (Nec-1s) blocks progression of existing aneurysms. (**A**) Mice were subjected to elastase perfusion. Starting on Day 7 post-elastase perfusion, mice received daily intraperitoneal injection of DMSO, Necrostatin-1 (Nec-1, 3.2 mg/kg/day), or Nec-1s (1.6 mg/kg/day), respectively. Mice were euthanized on Day 14. (**B**) Representative photos of abdominal aortae with indicated treatments. (**C**) Aortic diameters are expressed (left) as the percentage increase of maximal external aortic diameter between pre-perfusion and on Day 14 and (right) in millimeter measured on Day 14. An AAA is defined as a percentage increase in aortic diameter ≥100% (red dashed line). Kruskal–Wallis nonparametric test. Data represent mean ± SEM.

As shown in Fig. 5A, 7 days after elastase perfusion, we randomly divided mice to three groups, which received daily IP injections of either DMSO (post-elastase + DMSO), Nec-1 (3.2 mg/kg/day, post-elastase + Nec-1) or Nec-1s (1.6 mg/kg/day, post-elastase + Nec-1s) for the remaining 7 days of the study. Upon sacrifice, 14 days after elastase perfusion, mice in the post-elastase + DMSO group exhibited a mean aortic expansion of 172.80 ± 13.68% (1.408 ± 0.068mm) as compared to the day 7 measurement of 100.70 ± 4.37%, indicating progression of aneurysm growth. Mice treated with Nec-1 post-elastase displayed the mean aortic expansion of 121.60 ± 10.40% (1.133 ± 0.052mm), which was smaller than mice treated with DMSO, though this difference was not statistically significant (*P* = 0.1, Kruskal–Wallis nonparametric test; Fig. 5B,C). However, post-elastase treatment with Nec-1s (64.12 ± 4.80%; 0.864 ± 0.032 mm) significantly reduced aortic expansions compared to the post-elastase + DMSO group (*P* < 0.05, Kruskal–Wallis nonparametric test, Fig. 5B,C).

**Figure 6:**
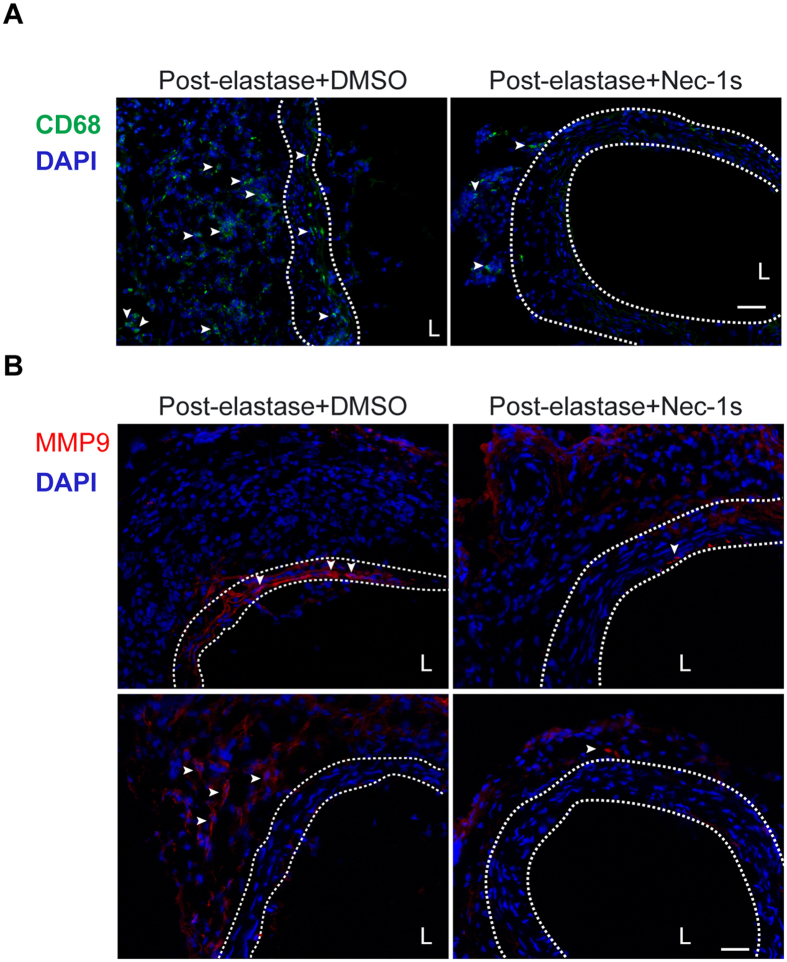
Necrostatin-1s (Nec-1s) treatment decreases macrophage infiltration and MMP9 accumulation in the elastase-injured aortae. Mice were subjected to elastase perfusion and started to receive daily intraperitoneal injection of DMSO or Nec-1s (1.6 mg/kg/day) from day 7 post-elastase perfusion. Mice were euthanized on Day 14. Representative photographs of immunostaining for the macrophage marker CD68 **(A)** and MMP9 **(B)** in the elastase-perfused aortae with indicated treatments. In (**B**), the top and bottom panels exemplify MMP9 expression in the media and adventitial aortic layers, respectively. Arrowheads indicate cells with positive CD68 or MMP9 staining. DAPI was used to stain the nuclei. Medial layer is highlighted by white dashed line. L indicates lumen. Scale bars = 50 μm.

We next evaluated whether daily administrations of Nec-1s could resolve the inflammatory response associated with the elastase-perfused aortae. Figure 6A demonstrates a marked reduction in macrophage infiltration in the elastase-perfused aortae, particularly in the medial layer, harvested from post-elastase + Nec-1s-treated mice. MMP9, produced primarily by inflammatory cells, plays important roles in aneurysm pathogenesis^38^. Consistent with the reduction of macrophage infiltration, immunostaining analysis revealed that MMP9 was reduced in both medial and adventitial layers of aortae from post-elastase Nec-1s-treated mice (Fig. 6B).

### Effects of Nec-1s on cytokine expression and macrophage migration

We recently demonstrated that the necroptotic mediator RIP3 affects the inflammatory response of SMCs through mechanisms that are both dependent and independent of cell death^20^. To clarify whether inhibition of RIP1 could reduce inflammation independent of its anti-cell death activity, we subjected MOVAS, a mouse aortic SMC cell line, to treatment with a low dose of TNFα (10 ng/ml, 2 hours or 2 ng/ml, 4 hours). We have previously established these stimulatory protocols to elicit optimal induction of different inflammatory genes without causing significant cell death^20^. As expected, TNFα significantly induced expression of *Ccl2 (MCP-1*), *Il*6*, Ccl*5*, Vcam*1 *and Icam*1 (Supplementary Fig. S2A). However, inhibiting RIP1 kinase with 20 μM Nec-1s, did not alter the upregulated expression of these cytokines or adhesion molecules triggered by the low doses of TNFα (Supplementary Fig. S2A). Similarly, in a mouse macrophage cell line RAW 264.7, LPS-induced MCP-1 and IL-6 expression was not affected by Nec-1s treatment (Supplementary Fig. S2B). To confirm that Nec-1s treatment does indeed inhibit RIP1 kinase activity at this concentration, we treated MOVAS with 20 μM Nec-1s and then stimulated the cells to undergo necroptosis using the TNFα plus zVAD protocol described earlier. At this concentration, Nec-1s significantly blocked necroptosis (Nec-1s: 6.75 ± 0.26% *vs*. DMSO: 45.15 ± 4.45%; Supplementary Fig. S2C). Futhermore, we tested whether Nec-1s affects monocyte/macrophage chemotaxis using a transwell assay. Surprisingly, pre-treatment of RAW 264.7 with 20 μM Nec-1s completely blocked cell migration toward MCP-1. The MAP Kinase inhibitor PD98059 was included as a positive control for migration blockage (Supplementary Fig. S2D).

### Nec-1s treatment promotes connective tissue repair in elastase-induced AAAs

**Figure 7:**
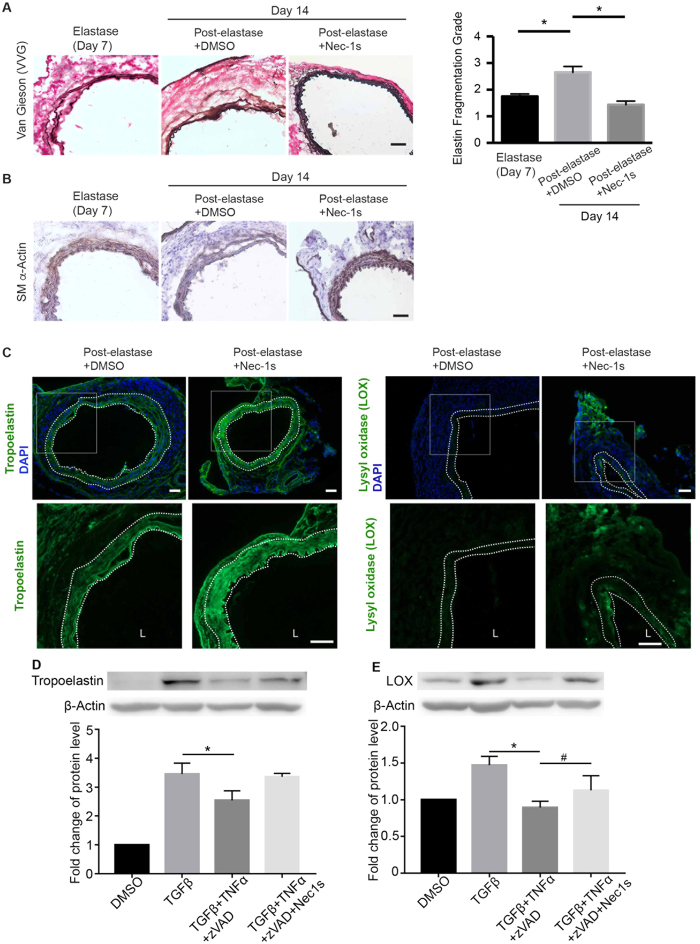
Necrostatin-1s (Nec-1s) treatment promotes aortic tissue repair. **(A)** Cross sections of harvested aortae were stained with Verhoeff-Van Gieson in which elastin fibers are stained in black (representative photos are shown in left panel). Scale bar = 50 μm. Fragmentation of elastin was graded according to an arbitrary scale of 1–4 as described in methods. Higher grading indicates more severe fragmentation. Quantification of the grading is shown on the right. Data are mean ± SEM. n = 5. *P < 0.05. (**B,C**) Representative photographs of immunostaining for the smooth muscle cell marker SM-αActin **(B)**, tropoelastin **(C**, left) and lysyl oxidase **(C**, right) in the elastase-perfused aortae (Day 14) with indicated treatments. In **C**, bottom images represent increased magnification of highlighted region in the upper images. DAPI was used to stain the nuclei. Medial layer is highlighted by white dashed line. L indicates lumen. Scale bars = 50 μm. (**D,E)** Primary mouse aortic SMCs were starved in 0.5% FBS low glucose DMEM for 24 h, then treated with TGFβ (10 ng/ml) for 48 h, and challenged with TNFα (20 ng/ml) plus zVAD (40 μΜ), Nec-1s (20 μM) for 6 h. Protein levels of tropoelastin and lysyl oxidase were detected by Western blot. n = 3. One-way ANOVA. *P < 0.05; ^#^*P* < 0.05.

AAA progression involves an imbalance between aortic tissue degeneration and repair^39^,^40^. Although the elastase-perfusion AAA model is acute compared to the chronic nature of human aneurysms, this mouse model recapitulates a progressive loss of aortic SMCs and elastin organization as reported previously^19^,^32^. Accordingly, the post-elastase + DMSO group exhibited the time-dependent tissue deterioration, evidenced by the more prominent elastin degradation and SMC depletion at Day 14 than at Day 7 (Fig. 7A,B). In contrast, mice treated with Nec-1s post-elastase perfusion displayed surprisingly normal looking arterial structure. A semi quantification of elastin fibers comparing elastase (day 7) and post-elastase + Nec-1s groups showed no progressive elastin degradation (Fig. 7A). The SM-αActin positive areas also appeared to be thicker and more intense in the sections from post-elastase + Nec-1s-treated mice (Fig. 7B). To further evaluate tissue repair, we examined the expression of tropoelastin and lysyl oxidase (LOX). As shown in Fig. 7C, Nec-1s treatment produced higher levels of aortic accumulation of tropoelastin and LOX. Moreover, in the primary cultured SMCs, we showed that a 6 h treatment with TNFα plus zVAD diminished TGFβ-induced production of LOX and tropoelastin. Nec-1s reversed the reduction of LOX and tropoelastin (Fig. 7D,E).

## Discussion

Loss of smooth muscle cells is a prominent pathological characteristic of abdominal aortic aneurysms^41^. Our results revealed in principle that inhibition of RIP1 slows aneurysm progression by reversing SMC loss, disrupting vascular inflammation and promoting synthesis of elastin. These findings also suggest that necroptosis and apoptosis have different roles in aneurysm pathophysiology, although both death mechanisms are detected in aneurysm tissues. We speculate that apoptosis is prominent in the initial phases of aneurysm development while necroptosis contributes more prominently during the later, progressive phases of AAA. Indeed, necroptosis appears to peak later than apoptosis in the elastase-perfusion model. However, it is important to note that aneurysmal aortic tissues derived from patients with advanced aneurysm show both apoptosis and high expression of the necroptosis mediators RIP1/3^18^,^20^.

A growing body of experimental evidence has implicated necroptosis, a form of regulated necrosis, in various cardiovascular diseases, including myocardial infarction^42^, atherosclerosis^43^,^44^, and AAA^20^. Necroptosis is characterized by its proinflammatory nature and its non-caspase mediators represented by RIP3 kinase, RIP1 kinase and the RIP3 substrate, mixed lineage kinase domain-like (MLKL)^23^,^45^,^46^,^47^. We have recently demonstrated that RIP3 expression in the arterial wall is required for elastase-induced AAA formation^20^. However, we are unable to examine how RIP3 inhibition affects existing aneurysms, since the currently available RIP3 kinase inhibitors have high cellular toxicity^25^. Both RIP1 and RIP3 are found to be upregulated in aneurysmal tissues^20^, however, the role of RIP1 inhibition on aneurysm pathophysiology cannot be simply extrapolated from the *Rip3* knockout studies^34^,^48^,^49^,^50^. Different and even opposite functions of RIP1 and RIP3 have been reported in certain disease models such as ethanol-induced liver injury^51^ or heart failure induced by ischemia-reperfusion or by oxidative stress^52^. Data presented here and from our previous study^20^ suggest that both RIP1 and RIP3 contribute to aneurysm pathogenesis via necroptosis. Interestingly, currently available RIP3 kinase inhibitors can cause profound apoptosis^25^ while this pro-apoptosis side effects are not seen in Nec-1 or Nec-1s treated cells. In this study, we observed that the Nec-1 or Nec-1s treatment was well-tolerated in the mice. The absence of apparent adverse effects of Nec-1 and Nec-1s is consistent with previous *in vitro* and *in vivo* studies using these inhibitors^29^,^30^,^53^ as well as the phenotype of mice baring the kinase inactivate RIP1 mutation^54^. In our study, inhibition of RIP1 with Nec-1 significantly reduced the number of necrotic cells as well as apoptotic cells in the elastase-perfused arteries. In cultured vascular SMCs, inhibition of RIP1 with the more selective inhibitor Nec-1s or an siRNA protected cells against necroptosis, suggesting the necessity of RIP1 in necroptosis of SMCs. In contrast, RIP3 gene deletion only diminishes necrosis without affecting apoptosis^20^,^27^. Another divergence between RIP1 and RIP3 is their complex interactions with the inflammatory pathway. We have shown in SMCs that RIP3 contributes to inflammation through both death-dependent and -independent mechanisms^20^, while RIP1 appears to be dispensable to the death-independent expression of pro-inflammatory cytokines in the aortic SMCs and macrophages. However, we found Nec-1s inhibit monocyte/macrophage chemotaxis, an effect that could contribute in part to the diminished inflammatory infiltration in Nec-1s treated mice. We also detected increased abundance of CD206+ cells (a marker for M2 or anti-inflmmatory macrophages^37^) relative to CD68+ cells in aortae following Nec-1s treatment, suggesting Nec-1s treatment have effects on macrophage polarization in aneurysmal tissues. Considering the complexity of macrophage phenotypes, in the future studies more M2 as well as M1 (proinflammatory) macrophage makers are needed to understand how Nec-1s treatment regulate macrophage phenotype in AAA. Moreover, Nec-1s treatment decreased the infiltration of T cells (CD3+) in the aneurysmal tissues. Those inflammation inhibitory effects of Nec-1s are likely to be indirect and depend on its ability to inhibit proinflammatory cell death. Although it has been well accepted that necrotic cells can trigger inflammation by massive release of immunogenic cell contents also called damage-associated molecular patterns (DAMPs)^55^, future studies need to validate and characterize the role of DAMPs *in vivo* by using antibody neutralization or gene deletion strategies.

The off-target of Nec-1 indoleamine 2,3-oxygenase (IDO), an immunomodulatory enzyme^56^, was reported to be essential for the suppressive effects of plasmacytoid dendritic cells on T-cell proliferation^57^. Through chemical modifications, Nec-1s is more potent and selective, and has no effects on IDO^56^. Nec-1s also shows improved stability *in vivo*^58^, which could in part explains why Nec-1s completely blocked the growth of existing aneurysms, a desirable effect that Nec-1 did not produce.

Aortic SMCs are a major cellular component of the arterial wall. SMCs regulate vascular tensile strengthen through synthesis of extracellular matrix (ECM) proteins including elastin and collagens^59^. Tropoelastin serves as elastin precursor while LOX initiates covalent crosslinking and deposition of collagen and elastin fibers. Huffman *et al*. discovered an insufficient vascular tissue repair process during elastase-induced AAA development. This process is featured by a transient upregulation of tropoelastin and progressive decrease of LOX^40^. Importantly, adenoviral vector mediated-overexpression of tropoelastin in the arterial wall inhibits elastase-induced aneurysm formation in rats^60^. Adenoviral expression of LOX ameliorates AAA progression in CaCl_2_ model^39^. We found in this study that post-elastase Nec-1s treatment elevated both tropoelastin and LOX in the elastase-perfused aortae. Interestingly, elevated tropoelastin primarily localized in the SMC-rich media while increased LOX expression was noted in both media and adventitia. The aortic elevation of tropoelastin and LOX by Nec-1s may be secondary to cell death inhibition, which results in a larger and healthier population of aortic SMCs available to direct tissue repair. In line with this argument, SMC-specific gene deletion of HIF-1a was recently reported to exacerbate aneurysm formation by suppressing synthesis of tropoelastin and LOX^61^. Alternatively, Nec-1s may target a regulator in the matrix metabolism pathway. However, Nec-1s was found to be very specific to RIP1. In a screen of more than 400 human kinases, Nec-1s displayed an over 1000-fold higher selectivity for RIP1^56^,^58^. Regardless of the underlying mechanism, Nec-1s not only saved SMCs but may also activated connective tissue repair during AAA progression, at least in the mouse elastase model.

**Figure 8:**
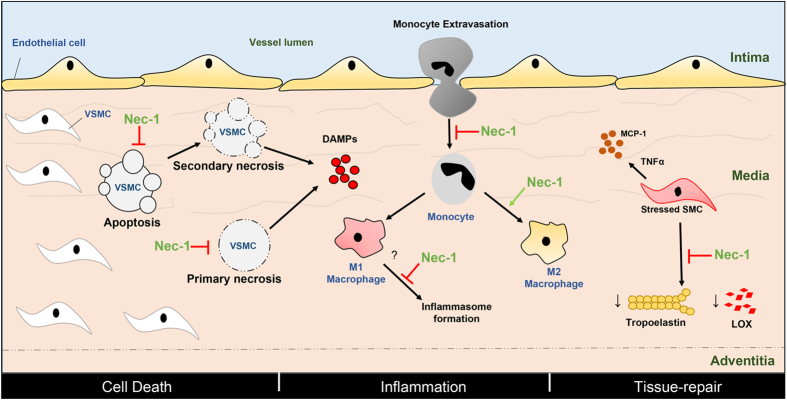
Proposed actions of Necrostatin-1 and its optimized form Nec-1s during the development and progression of abdominal aortic aneurysm. The common features of aneurysms– cell-death, inflammation and degradation of the extracellular matrix– are depicted in the vessel wall that consists of the intima, media and adventitia. VSMC in the media undergo apoptosis, primary or secondary (post-apoptotic) necrosis causing the release of DAMPs that subsequently stimulate inflammation. Nec-1/Nec-1s prevents apoptosis and necrosis, thereby reducing inflammation indirectly. Nec-1/Nec-1s may also directly reduce inflammation by preventing monocyte migration, inflammasome formation and increasing monocyte to M2 macrophage differentiation. Nec-1/Nec-1s may also promote tissue-repair through rescuing the reduction in the tropoelastin and LOX normally observed in the stressed VSMCs and therefore preserve extracellular matrix. Red arrows depict inhibition whereas green arrows show activation. VSMC: vascular smooth muscle cell, DAMP: damage-associated molecular patterns, LOX: lysyl oxidase, MCP-1: monocyte chemoattractant protein-1, TNFα: tumor necrosis factor alpha.

Based on data presented here, we propose Nec-1 and its optimized form Nec-1s attenuate development and progression of AAA by affecting cell death, inflammation, and ECM degradation (Fig. 8). SMCs undergo apoptosis, primary or secondary (post-apoptotic) necrosis causing the release of DAMPs that subsequently stimulate inflammation. Nec-1/Nec-1s prevents apoptosis and necrosis, thereby reducing inflammation indirectly. Nec-1/Nec-1s may also directly reduce inflammation by preventing monocyte migration and increasing monocyte to M2 macrophage differentiation. Moreover, Nec-1/Nec-1s could potentially promote tissue-repair through rescuing the reduction in the tropoelastin and LOX normally observed in the stressed VSMCs and therefore preserve extracellular matrix. While our data suggest Nec-1s may be considered as a potential AAA therapy, either alone or in combination with MMP inhibitors, results from mouse studies should be cautiously viewed as preliminary when dealing with complex human diseases. Although the mouse aneurysm models recapitulate major pathological features of human AAAs^19^,^32^, major limitations exist in aneurysm modeling. For one, AAA is a chronic disease. In patients, initiation, development, progression, and eventual rupture of aneurysms take place over the course of many years. In contrast, the current chemical-induced AAA mouse models produce a relatively acute injury that induces aortic inflammation and leads to aneurysm formation in weeks. Therefore, future studies should be directed to testing Nec-1s in more clinical relavent models such as aortic SMCs isolated from human aneurysmal tissues and in large animals.

To summarize, through the use of RIP1 kinase inhibitors, we demonstrate that aortic SMC death is an active component of aneurysm pathophysiology. Moreover, an optimized form of RIP1 kinase inhibitor Nec-1s blocked disease progression in mice with pre-existing small AAAs. While translating discoveries made in mice to clinical application is a very long journey, the proof-of-principle data obtained with Nec-1s along with elevated levels of RIP1 and RIP3 in human AAAs^20^ warrant future investigations to translate necroptosis inhibition to an aneurysm therapy.

## Materials and Methods

### General materials

Dulbecco’s Modified Eagle Medium (DMEM) was from Gibco (Life Technologies, Carlsbad, CA). Recombinant mouse TNF-alpha, TGF-beta 1 and MCP1 were from R&D Systems (Minneapolis, MN). Z-VAD-FMK was from Bachem (Torrance, CA). Necrostatin-1 was from Sigma (St. Louis, MO). Necrostatin-1s (7-Cl-O-Nec-1) was from BioVision (Milpitas, CA). Mitogen-activated protein kinase (MAPK) inhibitor PD98059 was from Biomol (Plymouth Meeting, PA). Primary antibodies used include anti-alpha smooth muscle actin, anti-tropoelastin, anti-lysyl oxidase (LOX), rabbit IgG (Abcam, Cambridge, MA) and anti-CD68 (AbD Serotec, Raleigh, NC). Fluorophore-conjugated secondary antibodies and 4′6-diamidino-2-phenyl-indole, dihydrochloride (DAPI) were purchased from Molecular Probes (Life Technologies, Carlsbad, CA). Horseradish Peroxidase (HRP)-conjugated Antibodies were purchased from Bio-Rad (Hercules, CA). *In Situ* Cell Death Detection Kit purchased from Roche Applied Science (Indianapolis, IN). Other chemicals and reagents if not specified were purchased from Sigma-Aldrich (St. Louis, MO).

### Mice

C57BL/6 J mice were purchased from The Jackson Laboratory (Bar Harbor, Maine). Male mice age 8–12 weeks old were used for experiments. All animal experiments were approved by the Institutional Animal Care and Use Committee at the University of Wisconsin-Madison (Protocol M02284). The procedures were carried out in accordance with the approved guidelines.

### Cell culture

The mouse aortic SMC cell line MOVAS cells and the mouse macrophage cell line RAW 264.7 cells were obtained from American Type Culture Collection (ATCC, Manassas, VA) and grown as recommended in DMEM modified containing 4.5 g/L D-Glucose (Life Technologies, Carlsbad, CA) supplemented with 10% FBS, 100 U/mL penicillin, and 100 U/mL streptomycin.

### Elastase-induced murine abdominal aortic aneurysm (AAA)

Male mice aged 8–12 weeks underwent an elastase-induced AAA model as described previously^16^,^19^,^62^. After anesthesia, the mouse abdomen was shaved and scrubbed with povidone-iodine (Betadine). The mouse was then placed supine on the operative field. A long midline abdominal incision was made and the abdominal aorta, extending from where the left renal vein crosses the aorta to the iliac bifurcation, was isolated. The external diameter of the largest portion of abdominal aorta was measured with a digital caliber. All of the aortic branches between the renal arteries and bifurcation were ligated with 9–0 sutures. After placing temporary 6–0 silk ligatures around the proximal and distal aorta, an aortotomy was created with a 30 G needle at the bifurcation. A heat-tapered segment of PE-10 polyethylene tubing (Baxter Healthcare Corp., Deerfield, IL) was introduced through the aortotomy and secured with a silk ligature. 0.45 U/mL type I porcine pancreatic elastase (Sigma, St. Louis, MO) was administrated to the infrarenal aorta through the tubing and allowed to incubate for 5 min at a constant pressure of 100 mm Hg. The 100mm Hg was calibrated using a saline bag hung at a height of 136 cm. As a control, a separate group of mice were treated with equal concentration of heat-inactivated (100 °C for 15 min) elastase for 5 min at a constant pressure of 100 mm Hg. The heat-tapered PE-10 polyethylene tubing was then removed and the aortotomy was closed with a 10–0 suture. The distal ligature was removed, followed by the removal of the proximal ligature. The abdominal incision was closed in two layers with 6–0 nylon sutures. Lidocaine 1% drops were placed on the wound. The mouse was kept on a warming pad until fully recovered from anesthesia. After the operation, mice were monitored each hour for the first 4 hours and then once daily. The external diameter of the largest portion of an abdominal aorta was measured with a digital caliper (VWR Scientific, West Chester, Pa) and recorded at sacrifice, as it had been prior to elastase perfusion. The percentage increase in maximal external aortic diameter was calculated.

Mice that underwent surgery were anesthetized using an isoflurane inhalant anesthetic: isoflurane was initially delivered via a chamber at 4%, followed by a mask at 2% isoflurane mixed with 100% oxygen. Mice were euthanized with 100% oxygen/5% isoflurane.

### Angiotensin II (Ang II)-induced murine abdominal aortic aneurysm (AAA)

Male, 12-week-old, apolipoprotein E–deficient mice were obtained from Jackson Laboratories (Bar Harbor, Me). Ang II (1000 ng/kg/min) was administered subcutaneously by Alzet osmotic minipumps (Alzet model 2004, Cupertino, Calif) for 28 days^17^,^63^. Mice were randomly grouped to receive either Necrostatin-1s (1.6 mg/kg/day) or DMSO via daily intraperitoneal injection immediately following minipump implantation. The external aortic diameter was measured at the region showing maximum dilation with a digital caliper. Aneurysm incidence is defined as an increase of 50% or greater in the external width of the suprarenal aorta as compared with that of the infrarenal region.

### RNA Isolation and Real-Time PCR (RT-PCR)

Total RNA was extracted from cultured cells using Trizol reagent (Life Technologies, Carlsbad, CA) according to the manufacturer’s protocols. Two micrograms total RNA was used for the first-strand cDNA synthesis (Applied Biosystems, Carlsbad, CA). RT-PCR was carried out using the 7500 Fast Real-Time PCR System (Applied Biosystems, Carlsbad, CA). Each cDNA template was amplified in triplicate using SYBR Green PCR Master Mix (Applied Biosystems, Carlsbad, CA) with gene specific primers. Primers for RT-PCR were QuantiTect Primers purchased from Qiagen (Valencia, CA). The relative mRNA levels were calculated using the 2^−ΔΔCT^ method. GAPDH was used as the endogenous control.

### Flow cytometric analysis

Cell death was evaluated by using an Annexin V-PE/7-AAD staining Kit (BD Biosciences, San Jose, CA). Cultures were rinsed with ice-cold PBS and incubated with accutase (Life Technologies, Carlsbad, CA) at 37 °C for 2 min. The detached cells (from culture medium, PBS wash, and accutase treatment) were collected by centrifugation (2000 rpm, 5 min). Cell pellets were further washed twice with ice-cold PBS and resuspended in 100 μl 1× binding buffer from the Annexin V-PE/7-AAD staining Kit. 5 μl of PE Annexin V and 5 μl of 7-AAD were added to the cells and incubated at room temperature for 15 min. After incubation, 400 μl binding buffer was added to each sample. Cells were analyzed using a Becton Dickinson Biosciences FACSCalibur (BD Biosciences, San Jose, CA).

### *In vivo* Propidium Iodide (PI) staining

*In vivo* cell necrosis was examined by intraperitoneal (IP) injection of propidium iodide (PI)^30^,^33^. PI (15 mg/kg body weight) was administered to mice through IP injection. Two hours after PI administration, mice were euthanized and perfusion fixed with 4% formaldehyde. 6-μm-thick cryosections were cut. Mounting medium with DAPI was applied before fluorescence microscopy examination. Staining was immediately visualized with a Nikon Eclipse Ti inverted microscope system and digital images were acquired using a Nikon DS-Ri1 digital camera.

Semi-quantification analysis of PI-positive cells in diseased tissues were performed as previously described^30^. PI-positive cells were counted in a blind fashion. The area of tunica media was measured with ImageJ (National Institute of Health, Bethesda, MD). At least five non-serial cross-sections per aorta were analyzed (n = 3 aortae per treatment).

### Immunohistochemistry

Aortas were perfusion-fixed with a mixture of 4% formaldehyde in phosphate buffered saline (PBS) under physiological pressure in order to preserve the structural morphology. Tissues were then harvested imbedded in O.C.T. Compound (Sakura Tissue Tek, Netherlands) and sectioned to 6 μm thickness using a Leica CM3050S cryostat. Tissue sections were permeabilized with 0.1% Triton X-100 in Tris-buffered saline (TBS) for 10 minutes at room temperature. Non-specific sites were blocked using 1% bovine serum albumin (BSA), 10% normal donkey serum in TBS for 2 hours at room temperature. Primary antibodies diluted in TBS with 1% BSA were then applied onto arterial sections, and incubated overnight at 4 °C. On the second day, arterial sections were rinsed with TBS plus 0.025% Triton X-100, followed by incubating with fluorophore-conjugated secondary antibodies diluted in TBS with 1% BSA for 1 hour at room temperature. DAPI was used to stain the nuclei. Staining was visualized with a Nikon Eclipse Ti inverted microscope system and digital images were acquired using a Nikon DS-Ri1 digital camera.

Terminal deoxynucleotidyl transferase dUTP nick end labeling (TUNEL) and semi-quantification analysis of TUNEL-positive cells in the media of aneurysmal tissues were performed as previously described^16^,^34^. TUNEL-positive cells were counted in a blind fashion. The area of tunica media was measured with ImageJ (National Institute of Health, Bethesda, MD). At least five non-serial cross-sections per aorta were analyzed (n = 3 aortae per treatment).

### Elastin grading

Elastin in frozen arterial cross sections was stained by using Richard-Allan Scientific™ Elastic Stain kit (Thermo Scientific, Rockford, IL). The foci of elastic lamellae fragmentation within 1 microscopic field were evaluated and graded according to an arbitrary scale of 1–4. Briefly, a grade of 1 indicates no elastin fragmentation or subintimal fragmentation; 2, fragmentation involving up to one-third of the media; 3, fragmentation involving two-thirds of the media; and 4, full thickness fragmentation.

### Migration assay

Macrophages (RAW 264.7 cell line) were starved in 0.5% high glucose DMEM for 6 h, then pretreated with 20 μM Nec-1s, 50 μM MAPK inhibitor PD98095, or DMSO for 2 hours. 2 × 10^4^ cells were placed in the upper chamber of Costar 24-well transwell plates with 5 μm pore filters (Corning, NY). Medium containing 100 ng/ml MCP1 and 20 μM Nec-1s, 50 μM PD98095 or DMSO was added into the lower wells. After 16 hours, cells that migrated to the bottom of the membrane were fixed, stained, and counted.

### Statistical analysis

Data are presented as mean ± SEM. In comparisons of two treatment conditions, two-tailed Student *t* test was used for normally distributed data and Mann–Whitney nonparametric test for skewed data that deviate from normality. In comparisons of three or more treatment conditions, one-way analysis of variance with Bonferroni post hoc test was used for normally distributed data and Kruskal–Wallis nonparametric test for skewed data. Differences with *P* < 0.05 were considered statistically significant.

## Additional Information

**How to cite this article:** Wang, Q. *et al*. Inhibition of Receptor-Interacting Protein Kinase 1 with Necrostatin–1s ameliorates disease progression in elastase-induced mouse abdominal aortic aneurysm model. *Sci. Rep.* **7**, 42159; doi: 10.1038/srep42159 (2017).

**Publisher's note:** Springer Nature remains neutral with regard to jurisdictional claims in published maps and institutional affiliations.

## Supplementary Material

Supplemental Information
