# Supplementary material for: Inhibition of Receptor-Interacting Protein Kinase 1 with Necrostatin–1s ameliorates disease progression in elastase-induced mouse abdominal aortic aneurysm model
Source: Sci Rep. 2017 Feb 10;7:42159. doi: 10.1038/srep42159 (PMC5301478; doi:10.1038/srep42159)
Supplement: Supplemental Information [file srep42159-s1.pdf]

Supplementary Information

Inhibition of Receptor-Interacting Protein Kinase 1 with Necrostatin–1s ameliorates disease progression in elastase-induced mouse abdominal aortic aneurysm model

Qiwei Wang[1*#], Ting Zhou[1*], Zhenjie Liu[1,2], Jun Ren[1], Noel Phan[1], Kartik Gupta[1], Danielle M Stewart[1], Stephanie Morgan[1†], Carmel Assa[1], K. Craig Kent[1$], and Bo Liu[1]

[1]Department of Surgery, School of Medicine and Public Health, University of Wisconsin, Madison, Wisconsin, USA and [2]Department of Vascular Surgery, 2nd Affiliated Hospital School of Medicine, Zhejiang University, Zhejiang, China.

* These authors contributed equally to this work.

# Present address: Department of Cancer Biology, Dana-Farber Cancer Institute, Harvard Medical School, Boston, MA, 02215.

† Present address: Center for Interdisciplinary Cardiovascular Sciences, Brigham and Women's Hospital, Harvard Medical School, Boston, MA, 02115.

$ Present address: Department of Surgery, The Ohio State University, Columbus, OH 43210

[#] **Address correspondence to:**

Bo Liu, Ph. D.

Department of Surgery, University of Wisconsin Madison

1111 Highland Avenue, WIMR 5137, Madison, WI 53705

Tel: 608-263-5931, Fax: 608-262-3330

E-mail: liub@surgery.wisc.edu

Supplementary Table S1. Outcomes of the Angiotensin II model.

Supplemental Table S1. Outcomes of the Angiotensin II model

Treatment	Total mice	Premature Mortality[a]	AAA Incidence (%)[b]	AAA Diameter (mm)	Aortic Expansion (%)[c]
DMSO	12	1	90.5	1.571±0.1405[d]	121.1±16.44[d]
Nec-1s	12	3	33.3	1.036±0.1017[d]	55.40±13.08[d]

[a]Number of mice died within the first week during angiotensin II infusion. The early deaths were caused by thoracic aortic ruptures preceded by dissections. The mortality rates between the two treatment group are not statistical significant (Two-tailed Chi-square test, P=0.27, Nec-1s *vs* DMSO)

[b]Mice died before Day 28 were excluded.

[c]Maximal external diameter of suprarenal aorta expressed as percentage of the infrarenal diameter.

[d]Data are presented as mean±SEM (Unpaired student's t test, P< 0.01, Nec-1s *vs* DMSO)

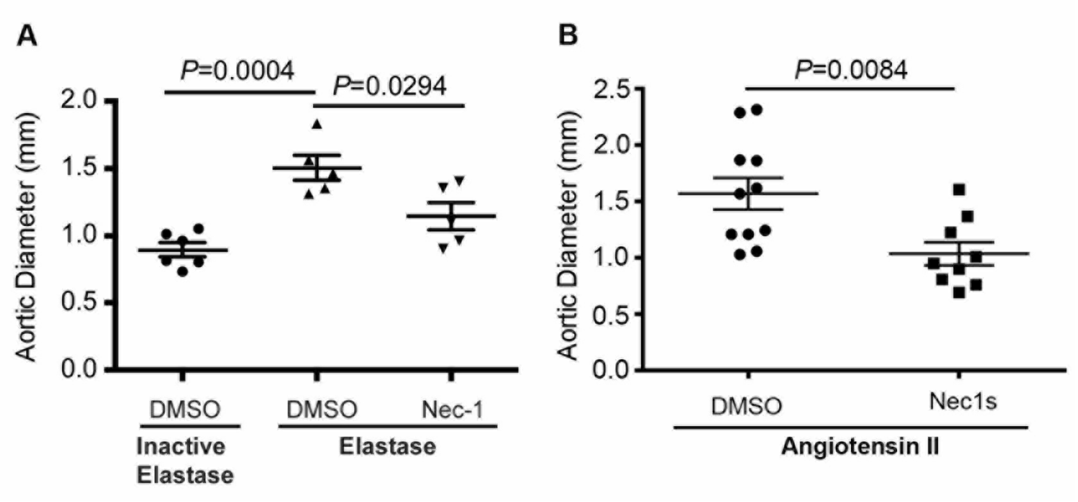

Supplementary Figure S1. Necrostatin 1 or 1s (Nec-1 or -1s) prevents aneurysm development. Maximal external aortic diameters in millimeter were measured and recorded at the end of elastase model (Day 14, **A**) and Ang II model (Day 28, **B**), respectively.

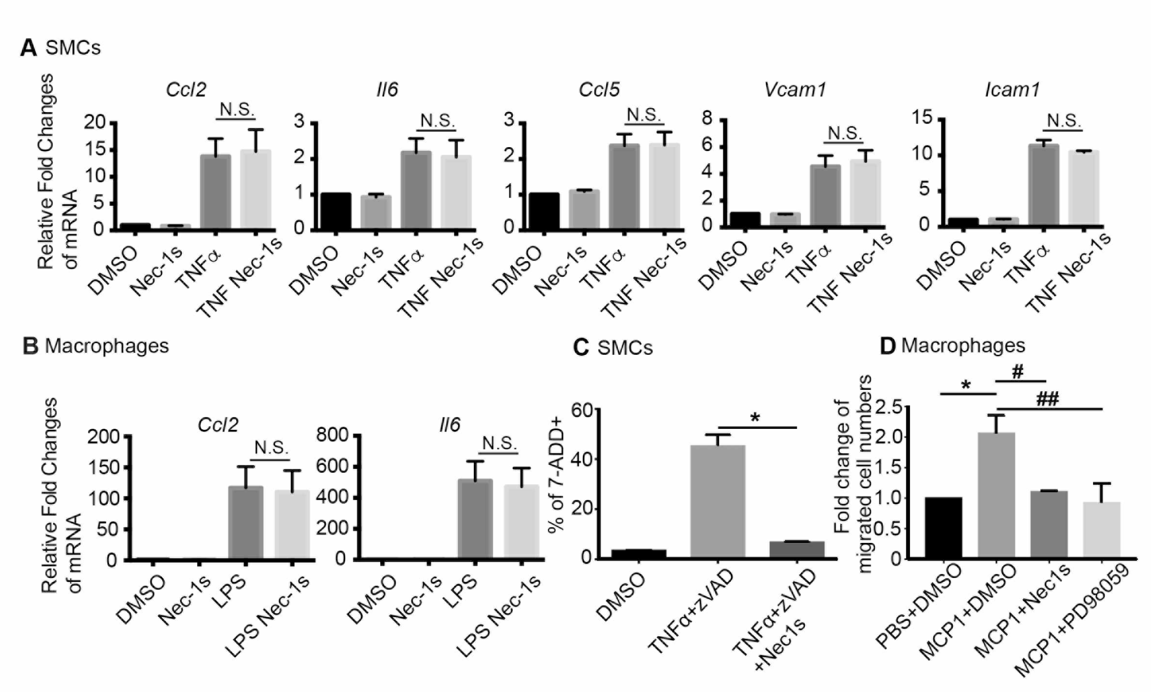

Supplementary Figure S2. Effects of Nec-1s on cytokine expression and macrophage migration. A, Mouse aortic SMCs were pre-treated with Nec-1s (20μM) for 1 hour. Cells were then incubated with DMSO or Nec-1s (20μM) in the presence or absence of TNFα (10ng/mL for 2 hours for cytokines or 2ng/mL for 4 hours for Vcam1 and Icam1). mRNA levels of indicated genes were determined by real-time PCR. **B**, Macrophages (RAW 264.7 cell line) were pre-treated with Nec-1s (20μM) for 1 hour. Cells were then incubated with DMSO or Nec-1s (20μM) in the presence or absence of LPS (100ng/mL) for 4 hours. mRNA levels of indicated genes were determined by real-time PCR. All values represent mean ± SEM. n=3. N.S.: not significant. **C,** MOVAS cells (a mouse SMC cell line) were challenged with TNFα (50ng/ml) plus zVAD (40μM) for 24 hours. RIP1 was inhibited with Nec-1s (20μM). Cell death was analyzed by flow cytometry following 7-AAD staining. All values represent mean ± SEM. n=3. *P<0.05. **D,** Macrophages (RAW 264.7 cell line) were pre-treated with Nec-1s (20 μM) for 2 h. 50μM MAPK kinase inhibitor PD98059 was used as a positive control. Cell migration toward MCP1 (100ng/ml) was determined by the transwell assay. n=3. One-way ANOVA. *P<0.05; # P<0.05 ; ##, P<0.01.
